# Attitudes toward psychopharmacology among hospitalized patients from diverse ethno-cultural backgrounds

**DOI:** 10.1186/1471-244X-8-55

**Published:** 2008-07-09

**Authors:** Gabriel Thorens, Marianne Gex-Fabry, Daniele F Zullino, Ariel Eytan

**Affiliations:** 1 Department of Psychiatry, Division of Substance Abuse, University Hospitals of Geneva, Switzerland; 2 Department of Psychiatry, Division of Adult Psychiatry, University Hospitals of Geneva, Switzerland; 3 Department of Psychiatry, Division of Penitentiary Psychiatry, University Hospitals of Geneva, Switzerland

## Abstract

**Background:**

Biological factors influencing individual response to drugs are being extensively studied in psychiatry. Strikingly, there are few studies addressing social and cultural differences in attitudes toward psychotropic medications. The objective of this study was to investigate ethno-culturally determined beliefs, expectations and attitudes toward medication among a sample of hospitalized psychiatric patients.

**Methods:**

An ad hoc questionnaire was designed to assess patients' expectations, attitudes and prejudice toward medication. The study included 100 adult patients hospitalized in Geneva, Switzerland.

**Results:**

Patients were in majority male (63%), originated from Switzerland (54%) and spoke the local language fluently (93%). They took on the average 3 different psychotropic drugs. Sixty-eight percent of patients expected side effects and 60% were ready to stop medication because of them. Thirty percent of patients expected negative personal changes with treatment and 34% thought that their mental disorder could have been treated without drugs. Thirty six percent of the sample used alternative or complementary medicines. 35% of immigrant patients believed that medication had different effects on them than on local patients. When compared with Swiss patients, they more often reported that significant others had an opinion about medication (p = 0.041) and more frequently valued information provided by other patients about treatment (p = 0.010).

**Conclusion:**

Patients' attitudes toward medication should be investigated in clinical practice, as specific expectations and prejudice exist. Targeted interventions, especially for immigrant patients, might improve adherence.

## Background

Biological factors determining the effects of psychotropic medications are extensively studied for each drug category, both from pharmacokinetic and pharmacodynamic perspectives. The study of genetic variants influencing drug metabolism has developed more recently, through the emergence of pharmacogenetics [1]. This approach might permit to draw different metabolic profiles for different ethnicities and races, even though its clinical use is still limited [2,3]. More broadly, the utilization of genetic information to predict outcome of drug treatment, or pharmacogenomics, holds great promise for clinical psychiatry [4].

However, non-biological factors such as level of adherence to medication continue to impact profoundly upon treatment outcome [5]. Medication nonspecific therapeutic and adverse effects, respectively the placebo and nocebo phenomena, constitute important determinants of treatment outcome [6]. Social and ethno-cultural determinants of drug treatment outcome, such as patients' expectations and attitudes toward psychotropic medication, remain understudied. In other words, clinicians and researchers seem to pay more attention to the biological side of pharmacotherapy than to contextual issues [7]. Patients' expectations about treatment effects may be influenced by pill size, shape, and color, among other factors. The often cited study by Buckalew and Coffield showed that capsules were perceived as stronger than tablets, larger capsules were seen as stronger than smaller ones, and some capsule colors were specifically ascribed expectations about drug action [8]. Important inter-ethnic variations were demonstrated, e.g. Caucasians expected black capsules to be stimulant, while African-Americans expected them to be sedative [9]. In the present article, the term ethno-cultural is used in its broad definition and has to do with collective identity, i.e. the setting apart of one group of people from another on the basis of historical lineage, language, religion and membership of a community [10].

Ethno-cultural differences have repeatedly been found with regard to attribution of causes of various disorders (e.g. somatic vs. spiritual/divine). These "explanatory models of illness" are of special importance in mental health [11]. Eventually, culture influences representations, compliance, expectations about treatment and engagement with services [12].

Some psychometric instruments have been developed in order to analyze subjective parameters of pharmacotherapy. To our knowledge, these instruments took into consideration ethnic and cultural factors only marginally. For example, the widely used Drug Attitude Inventory (DAI), which was translated into several languages since its initial validation in the eighties, does not investigate ethno-cultural aspects, neither in its shortened version (DAI-10), nor in its long version (DAI-30) [13]. Several other instruments exist, but they address exclusively psychotic disorders [14-16]. The aim of the present study was to contribute filling this gap and analyze cultural factors influencing attitudes toward psychotropic drug treatment in a multi-ethnic sample of psychiatric inpatients.

## Methods

### Setting

The study was conducted in one admission unit of the public psychiatric hospital in Geneva, an international, multicultural city of approximately 430'000 inhabitants located in the French speaking part of Switzerland. During the study period, the 15 to 18-bed general psychiatry unit received patients with primary diagnoses of affective disorders (33%), schizophrenia and other psychotic disorders (33%), alcohol and other substance use disorders (15%), personality disorders (10%) and other disorders (9%). During the 11-month recruitment period, a total of 400 patients were hospitalized in the unit (mean length of stay 14.4 days). All patients admitted to this unit and meeting inclusion criteria were individually approached. Inclusion criteria were sufficient length of stay to allow for psychiatric assessment (at least 2 days) and for partial remission of acute behavioral or psychotic symptoms. Patients who gave written informed consent were interviewed until 100 participants were recruited. Patients who did not provide informed consent did not differ clinically from those who gave consent. The study was approved by the local ethics committee.

### Questionnaire

Taking into account a literature review about cultural considerations in non-biological issues affecting psychopharmacotherapy [17], we developed an ad hoc observer-rating questionnaire. Three preliminary versions were pre-tested, thus permitting to reformulate or eliminate questions which were too difficult to understand or too vague. In its final version (available upon request), the questionnaire contained 22 items. Answers were rated on Likert scales (e.g. "not at all", "not really", "not sure", "rather yes", and "yes, very much"). For analysis, values were dichotomized by grouping the 3 former categories and the 2 latter ones, respectively. The following domains were investigated: personal opinion about drug aspect, administration mode and information about treatment, expected drug effects, use of alternative or complementary medicine and perceived influence of context and general attitude toward medication. Finally, four questions specifically addressed beliefs among immigrants to Switzerland.

Two experienced clinicians (a psychiatrist and a psychologist) conducted the interviews. Professional translators were used for patients who did not speak French, the local language.

### Patients

One hundred hospitalized patients were included in the study (age range 19–65). Median length of stay was 7 days (range 2–67). Median number of prescribed drugs at the moment of the interview was 3 (range 0–10).

Patients were pooled into 3 groups according to their country of origin:

(1) 54 patients originated from Switzerland

(2) 25 patients came from a country that was part of the European Union (EU) (Italy n = 6, Spain n = 6, Portugal n = 6, France n = 5, Greece n = 1, UK n = 1)

(3) 21 patients came from non-EU countries (Turkey n = 3, Zaire n = 2, Algeria, Angola, Bosnia, Brazil, Burundi, Cameroon, Chile, Congo, Morocco, Nigeria, Poland, Rumania, Rwanda, Senegal, Sudan, USA: n = 1 from each country). Poland and Rumania were not yet EU members at the time of the study.

The first group corresponds to patients sharing the local culture; the second group corresponds to patients that share some European ethno-cultural characteristics; the third group is composed of persons with very diverse ethno-cultural backgrounds.

Among subjects with a non-Swiss origin, 5 (10.9%) were asylum-seekers, 3 (6.5%) had a non-permanent residence permit (type B permit, valid for five years), 24 (52.2%) had a permanent residence permit (type C, with unrestricted access to labor market) and 13 (28.3%) had become Swiss citizens.

Socio-demographic and treatment characteristics of participants are reported in Table 1.

**Table 1:** Socio-demographic characteristics and current pharmacotherapy

	**Swiss** **(N=54)**		**European** **Union** **(N=25)**		**Others** **(N=21)**		**p**
Women	21	38.9%	10	40.0%	6	28.6%	0.67
Age ≥ 40 years	26	48.1%	12	48.0%	4	19.0%	0.06
Religion							
Catholic	26	48.1%	19	76.0%	9	42.9%	0.001
Protestant	12	22.2%	0	0.0%	0	0.0%	
Jewish	0	0.0%	0	0.0%	0	0.0%	
Muslim	4	7.4%	0	0.0%	6	28.6%	
Atheist	8	14.8%	4	16.0%	2	9.5%	
Other	4	7.4%	2	8.0%	4	19.0%	
French skills							
Excellent	54	100.0%	22	88.0%	17	81.0%	0.006
Fair	0	0.0%	2	8.0%	2	9.5%	
Insufficient	0	0.0%	1	4.0%	2	9.5%	
Living status							
Living alone	26	48.1%	9	36.0%	11	52.4%	0.74
Couple	12	22.2%	6	24.0%	2	9.5%	
Family	11	20.4%	8	32.0%	6	28.6%	
Institution	5	9.3%	2	8.0%	2	9.5%	
Education (N = 99)							
None – elementary	4	7.4%	1	4.0%	0	0.0%	0.46
Compulsory education	12	22.2%	11	44.0%	6	30.0%	
Apprenticeship	20	37.0%	6	24.0%	5	25.0%	
College	7	13.0%	5	20.0%	4	20.0%	
Technical school	6	11.1%	1	4.0%	1	5.0%	
University	5	9.3%	1	4.0%	4	20.0%	
Current medication (N = 95)							
Antipsychotics	28	54.9%	11	47.8%	15	71.4%	0.26
Antidepressants	28	54.9%	13	56.5%	6	28.6%	0.10
Mood stabilizers	21	41.2%	4	17.4%	5	23.8%	0.09
Tranquilizers	18	35.3%	10	43.5%	6	28.6%	0.63
Hypnotics	20	39.2%	5	21.7%	8	38.1%	0.34

### Statistical analysis

The Fisher exact test for proportions was used for comparisons between groups, with significance level set at 0.05 (2-sided). Data were analyzed using the SPSS program, version 11 (SPSS Inc., Chicago IL, USA).

## Results

### Socio-demographic characteristics and current pharmacotherapy

As shown in Table 1, the 3 groups significantly differed with respect to religion, with Catholics over represented among patients from the EU, Protestants only represented among Swiss patients and the largest proportion of Muslims among non-EU subjects. Even though differences between groups were significant, a large majority of patients spoke French fluently in all 3 groups (> 80%). There were no significant differences between groups with regard to prescribed psychotropic medication.

### Patient opinion about drug aspect, formulation and information

A majority of patients rated the general aspect of the medication as important or very important. Taste was the specific aspect most often rated as important, followed by tablet size, shape, color, and package design. No significant difference was observed between patients of different origins (Table 2).

**Table 2:** Patients' opinion about drug aspect, formulation and information ^§^

	**Total ** **sample ** (N=92–100)*	**Swiss** (N=50–54)*	**European ** **Union** (N=21–25)*	**Others ** (N=20–21)*	**p**
**Are the following aspects of the drug important to you?**					
General aspect	54.5%	58.5%	44.0%	57.1%	0.53
Taste	56.0%	51.9%	56.0%	66.7%	0.53
Size	46.0%	46.3%	44.0%	47.6%	1.00
Shape	33.0%	37.0%	16.0%	42.9%	0.10
Color	31.0%	31.5%	20.0%	42.9%	0.25
Package design	26.0%	31.5%	8.0%	33.3%	0.047
					
**If possible, would you select the following administration modes?**					
Tablets	92.9%	90.6%	96.0%	95.2%	0.77
Effervescent tablets	62.6%	61.1%	58.3%	71.4%	0.70
Drops	45.9%	45.3%	50.0%	42.9%	0.89
Intravenous injection	26.5%	22.6%	33.3%	28.6%	0.56
Intramuscular injection	18.4%	18.9%	12.5%	23.8%	0.69
Subcutaneous injection	16.5%	23.1%	12.5%	4.8%	0.15
					
**From which sources do prefer to be informed about drug treatment?**					
Physician	92.6%	92.2%	95.7%	90.0%	0.88
Nurse	74.2%	74.0%	73.9%	75.0%	1.00
Written patient information	74.2%	80.4%	59.1%	75.0%	0.17
Books, magazines	32.6%	32.0%	31.8%	35.0%	1.00
Family, important others	26.1%	24.0%	27.3%	30.0%	0.86
Television	25.0%	17.6%	28.6%	40.0%	0.12
Other patients	13.0%	4.0%	22.7%	25.0%	0.010

* Missing values excluded from % calculation.

^§ ^Percents refer to patients who answered "yes" or "yes, very much"

If they could choose the administration mode of their psychotropic treatment, most patients would prefer to take the drug orally. Only a minority of patients (< 30%) would select intravenous, intramuscular or subcutaneous administration (Table 2).

A large majority of patients (≥ 90%), independently of their country of origin, would prefer to be informed about drug therapeutic and adverse effects by their attending physician. Information by nurses and written patient information were cited next. Interestingly, Swiss patients mentioned information from other patients significantly less often than foreigners (Table 2).

### Expected effects of medication and perceived influence of ethno-cultural context

Sixty-eight percent of patients expected side effects and 60% thought that adverse effects might be severe enough to let them stop treatment. Thirty percent expected negative personal changes with treatment, whereas 62% expected positive changes. Thirty six percent of the sample used alternative or complementary medicines. While the opinion of significant others about treatment was more frequently cited among patients from foreign countries than from Switzerland, no difference was found for the possible interference between religious beliefs and treatment (Table 3).

**Table 3:** Expected effects of medication and perceived influence of ethno-cultural context ^§^

	**Total** **sample** (N=99–100)*	**Swiss ** **(N=54)**	**European ** **Union ** (N=24–25)*	**Others ** **(N=21)**	**p**
Do you expect side effects?	68.0%	66.7%	60.0%	81.0%	0.29
Might these side effects be severe enough to let you interrupt the treatment?	60.0%	55.6%	56.0%	76.2%	0.25
Do you expect *positive *personal changes (character, personality ...) due to pharmacotherapy?	62.0%	64.8%	60.0%	57.1%	0.82
Do you expect *negative *personal changes (character, personality ...) due to pharmacotherapy?	30.0%	24.1%	40.0%	33.3%	0.32
Do significant others have an opinion regarding your treatment?	57.6%	46.3%	75.0%	66.7%	0.041
Do you use alternative/complementary medicine (homeopathy, traditional medicine etc.)?	36.4%	44.4%	33.3%	19.0%	0.12
Does your physician's attitude influence treatment result?	72.0%	66.7%	72.0%	85.7%	0.28
Does your nurses' attitude influence treatment result?	69.0%	66.7%	60.0%	85.7%	0.14
Do your religious beliefs and practices interfere with treatment?	9.0%	5.6%	16.0%	9.5%	0.32

* Missing values excluded from % calculation.

^§ ^Percents refer to patients who answered "yes" or "yes, very much"

### Patients' attitudes toward medication

Thirty four percent of patients thought that their mental disorder could have been treated without drugs. Patients from non-European countries were significantly less often positive about taking medication than patients from the other two groups. However, in each group, a large majority of patients (> 90%) reported that they would probably or certainly continue to take the medication as prescribed after discharge from the hospital.

When compared with patients from EU-countries, subjects from non-European countries significantly more often thought that they received medication not available in their home country. While more than 80% of EU-patients believed that they would have the same reaction to medication as Swiss patients, this was the case for only 45% of non-European patients (Table 4).

**Table 4:** Patients' attitudes toward medication ^§^

	**Total ** **sample** (N=99–100)*	**Swiss** (N=53–54)*	**European ** **Union ** (N=17–25)*	**Others** (N=17–21)*	**p**
Do you believe that you could have been treated without medication?	34.0%	29.6%	28.0%	52.4%	0.15
Is your self image modified by the medication you are taking?	48.5%	43.4%	56.0%	52.4%	0.55
What is your general opinion about taking medications? (% positive or very positive)	59.0%	68.5%	64.0%	28.6%	0.007
What is your general opinion about taking psychiatric medications? (% positive or very positive)	44.0%	51.9%	40.0%	28.6%	0.19
Are you going to take your medication as prescribed after discharge from the hospital? (% probably yes or yes)	94.0%	94.4%	92.0%	95.2%	0.86
Do you consider all medications the same way?	16.0%	16.7%	8.0%	23.8%	0.35
Do you receive a treatment that you could not get in your country of origin?			17.6%	58.8%	0.032
Could you receive, in your country of origin, a treatment you do not get here?			13.0%	31.6%	0.26
Do you think you get the same treatment as a Swiss patient?			78.3%	90.0%	0.42
Do you think you have the same reaction to medication as a Swiss patient?			82.6%	45.0%	0.013

* Missing values excluded from % calculation.

^§ ^Percents refer to patients who answered "yes" or "yes, very much", unless indicated otherwise

## Discussion

Globalization has led to a significant increase in migrations. People are now moving further, faster and in greater numbers than ever before [18]. This phenomenon impacts on psychiatric services. Several studies have indeed shown that patients consulting in western psychiatric facilities are increasingly multiethnic and multicultural [19]. In this context, it is important for caregivers to be aware of patients' attitudes toward treatments, as a form of cultural competence [20].

To our knowledge, the present study is the first European survey examining differences of attitudes and beliefs toward psycho-pharmacotherapy in psychiatric patients from various ethno-cultural backgrounds. Our study confirms the importance given by patients to external characteristics of drugs, like taste and tablet size, and their preference for oral administration. There were few intercultural differences in this respect.

Physicians were clearly the preferred information source for the whole sample. Foreign patients tended to rely more on other patients on the ward as a source of information, compared with Swiss subjects. One possible explanation is that Swiss patients are more familiar with the local medical system and therefore accept therapeutic procedures more easily than foreign patients, the latter needing reassurance from experienced persons outside the medical staff. It could also be caused by insufficient or inadequate medical information given to foreign patients. The appropriate transmission of medical information is dependent on communication between staff and patient. Both profound cultural differences and insufficient use of trained interpreters can impede such communication in our local setting [21].

The opinion of relatives about treatment was cited more frequently by foreign patients than local patients. This could be due to differences regarding familial cohesion, which can be considered as traditionally stronger in Southern and Eastern countries than in Western Europe or the US. Indeed, migrants included in this study moved from collectivist (or socio-centric) societies to an individualistic (or ego-centric) one [22]. This finding could also reflect an increased need for peer support among people living in a foreign country.

The general opinion of non-European patients about taking medications was less often positive than among the European sub-groups. This result might reflect differences in diagnoses and/or cultural representations: Western medicines may be subject to different beliefs by different cultural groups, affecting their attitudes toward them [23]. Strikingly, more than 90% of all participants declared that they intended to take their medication as prescribed after discharge from the hospital. This result contrasts with adherence rates usually reported in the literature: rates of adherence for psychiatric illnesses are seldom above 65% and sometimes as low as 35% [24]. Our result could reflect social desirability and willingness to appear adherent to treatment.

This study has several limitations. Firstly, country of origin was used as a proxy for ethno-cultural background. Place of birth and language spoken at home are more commonly accepted indicators [25]. However, since obtaining the Swiss nationality is a long and complicated process, we believe that most immigrants included in the study would have retained their nationality of origin. Secondly, attitudes toward medication were investigated from the patient's perspective only. It is however well known that patterns of prescription also vary with physicians' cultural stereotypes [26]. In the present study, although not statistically significant, non-European patients tended to receive more often antipsychotic medications and less often antidepressants than Swiss and European patients (p = 0.26 and 0.10 respectively). Whereas these different prescription patterns might be associated with different distribution of diagnoses across groups, a cultural bias could also be invoked. Thirdly, other cultural factors that may influence preferences and attitudes toward treatment, such as spirituality, stigma or access to care [27], were not investigated. Fourthly, diagnoses were not recorded. However, we found no significant differences between patients treated with or without antipsychotic medication. This observation indicates that patients with a diagnosis of psychotic disorder, i.e. approximately one third of the sample, did not differ from patients with other diagnoses regarding attitudes toward medication. Fifthly, due to the absence of appropriate instruments in the literature, we used a non-validated questionnaire designed purposely for the study. Finally, the sample was too small to demonstrate subtle differences between the 3 different cultural groups.

## Conclusion

Patients treated in a Swiss psychiatric hospital share most of their pharmacotherapy related attitudes and expectations independently of their region of origin. Medical staff members are the most trusted sources of information. However, there are some important differences between local and foreign patients. Results speak in favor of better integrating relatives of non-European patients in the therapeutic process. Sharing experiences, beliefs and expectations regarding treatment with other patients also appear to be of a special importance for foreign subjects. In order to improve adherence with treatment, these observations should be taken into account when organizing clinical services that serve culturally diverse populations.

## Competing interests

The authors declare that they have no competing interests.

## Authors' contributions

AE conceived the study and drafted the manuscript. GT coordinated the study and conducted some of the interviews. MGF participated in the design of the study and performed the statistical analysis. DFZ informed the discussion.

## Pre-publication history

The pre-publication history for this paper can be accessed here:


